# The Establishment of a CSF-Contacting Nucleus “Knockout” Model Animal

**DOI:** 10.3389/fnana.2018.00022

**Published:** 2018-03-27

**Authors:** Si-Yuan Song, Li-Cai Zhang

**Affiliations:** Jiangsu Province Key Laboratory of Anesthesiology, Xuzhou Medical University, Xuzhou, China

**Keywords:** CSF-contacting nucleus, knockout, model animal, CB-SAP, targeted ablation

## Abstract

To establish an entirely cerebrospinal fluid (CSF)-contacting nucleus-deficient model animal, we used cholera toxin B subunit (CB)- saporin (SAP), which is an analog of CB-HRP that specifically labels the CSF-contacting nucleus, to exclusively damage the nucleus. The effectiveness and specificity of the ablation were evaluated upon days 1–10 after CB-SAP microinjection into the brain ventricular system. The vital status, survival, and common physiological parameters of the model animals were also assessed during the experimental period. The results demonstrated that CB-SAP damaged only the CSF-contacting nucleus, but not other functional structures, in the brain. The complete ablation occurred by day 7 after CB-SAP microinjection. A model animal that had no CSF-contacting nucleus was established after survival beyond that time point. No obvious effects were observed in the vital status of the model animals, and their survival was ensured. The common physiological parameters of model animals were stable. The present study provides a method to establish a CSF-contacting nucleus “knockout” model animal, which is similar to a gene knockout model animal for studying this particular nucleus *in vivo*.

## Introduction

The brain-cerebrospinal fluid barrier is like a mysterious veil that blocks information transmission, substance exchange and functional modulation between the brain parenchyma and the cerebrospinal fluid (CSF). During several decades, the CSF-contacting structures were found near the walls of the cerebral ventricles and the central canal of the spinal cord. Vigh et al. regard the CSF-contacting neuron (CSF-CN) as a peculiar cell type within the central nervous system and hypothesized that it performs a pivotal role in non-synaptic signal transmission in the brain (Vigh-Teichmann and Vigh, 1989; Vígh et al., 2004). However, methodological limitations have confined almost all previous studies on CSF-contacting structures to sites near the walls of the cerebral ventricles and the central canal of the spinal cord. The location and distributional regularity of the CSF-CNs that exist within the brain parenchyma remain almost unknown.

Over the past 20 years, our research group has made great progress in labeling CSF-CNs and have proved that cholera toxin B subunit (CB) and CB-horseradish peroxidase (CB-HRP) specifically label CSF-CNs in the parenchyma (Wang and Zhang, 1992; Lu et al., 2008; Zhou et al., 2013). By applying these results, Zhang et al. successively reported the distribution of different types of CSF-CNs in the brain parenchyma (Zhang et al., 1994, 1995). In addition, reciprocal synaptic connections of the CSF-CNs with other neurons in the brain parenchyma and bidirectional non-synaptic communication with the CSF and blood vessels have been confirmed (Zhang et al., 2003; Liang et al., 2007). Later studies have repeatedly confirmed that the CSF-CNs in the brain parenchyma form a constant and intensive cluster within the ventral gray of the lower portion of the aqueduct and upper portion of the fourth ventricle floor. We name this independent neuron cluster the “cerebrospinal fluid-contacting nucleus” or “CSF-contacting nucleus” (Wang et al., 2013, 2014; Liu et al., 2014; Wu et al., 2014). A unique feature of the CSF-contacting nucleus is that its neural somas are located within the brain parenchyma. They can communicate with other structures in the brain, such as non-CSF-contacting neurons, glia cells, and blood vessels. Their processes also stretch into the CSF. They can transmit information between the brain parenchyma and the CSF or into the blood. Therefore, the CSF-contacting nucleus may play a pivotal role in modulating both the neuro-neural and the neuro-body fluid circuits. The CSF-contacting nucleus can even be regarded as a bridge or hinge that facilitates neuro-neural crosstalk and neuro-body fluid communication. However, the essential role of the CSF-contacting nucleus in regulating entire biological functions has not yet been clarified.

The “yes-no method” is one of the fundamental principles in signal detection theory. This method applies the gene knockout technique to make a specific gene deficiency (Rozas et al., 2012) or the degeneration technique to damage a specific neuron or nucleus (Thiele et al., 2014), thus establishing corresponding model animals for studying the biological functions of specific genes, neurons, or nuclei. The “yes-no method” has particular application in unveiling the existence of neural circuits or clarifying the functions of specific neurons or nuclei. Therefore, we decided to specifically damage the CSF-contacting nucleus and establish a model animal that has no CSF-contacting nucleus, which is similar to a gene knockout.

The key step in damaging the CSF-contacting nucleus is to identify an agent that specifically damages this nucleus, but not other structures, in the brain. Our research group has shown that CB-HRP (a tracer used mainly for the peripheral nervous system) specifically labels the CSF-contacting nucleus (Wang and Zhang, 1992; Lu et al., 2008; Zhou et al., 2013). We therefore hypothesized that CB-saporin (SAP), a degeneration agent used mainly for the peripheral nervous system, is likely to specifically damage the CSF-contacting nucleus because CB-SAP is an analog of CB-HRP. Additionally, the principle of degeneration is similar to that of tracing (Nichols et al., 2015).

The present study uses Sprague-Dawley rats as subjects. After CB-SAP microinjection into the ventricular system, the effectiveness and specificity of the ablation were assessed through different approaches. The animals' vital status, survival, and common physiological parameters were assessed after CSF-contacting nucleus ablation. The aim of this study is to provide a scientific and reliable CSF-contacting nucleus “knockout” model animal for researchers who are interested in this special nucleus within the brain.

## Materials and methods

### Experimental animals and CB-SAP microinjection

SPF grade Sprague-Dawley rats of both sexes (weight 250–300 g) were acquired from the Experimental Animal Center of Xuzhou Medical University. All experiments were approved and performed in accordance with the Committee for Ethical Use of Laboratory Animals, Xuzhou Medical University. Rats were anesthetized with pentobarbital sodium (40 mg/kg, i.p.), and their heads were fixed on a stereotaxic instrument (Stoelting 51700, USA). CB-SAP (ATS, USA) was dissolved in PBS (0.01 M, pH 7.4), and 3 μl (500 ng) CB-SAP was microinjected into the lateral ventricle according to the stereotaxic coordinates provided by Paxinos and Watson (2007). The injections into the lateral ventricle were confirmed by successfully extracting the CSF.

### Evaluation of the specificity of the CB-SAP ablation for the CSF-contacting nucleus

Rats were divided into three groups (A, B, and C). Group A was perfused at approximately 24 h after CB-SAP microinjection into the lateral ventricle, which corresponds to the length of time required for the CSF to finish circulating in the brain ventricular system. The brain and the spinal cord were sectioned coronally at 40 μm thickness (Leica CM1900, Germany) and underwent SAP immunofluorescence reaction (anti-SAP diluted in 1:400, ATS). The sections containing the CSF-contacting nucleus underwent CB/SAP immunofluorescent double staining (anti-CB diluted in 1:600, Merck Millipore; anti-SAP diluted in 1:400, ATS).

Group B was the ablation group that was perfused at 5 days after CB-SAP microinjection into the lateral ventricle to investigate the influence of the degeneration agent CB-SAP on the CSF-contacting nucleus and other structures in the brain that directly contact the CSF. These other structures include tanycytes, ependymal cells, and other ultrastructures within the ventricle wall as well as the DR, which is the nearest structure adjacent to the CSF-contacting nucleus in the parenchyma. A segment of the CSF-contacting nucleus was cut into slices and underwent a CB immunofluorescent reaction (anti-CB diluted in 1:600, Merck Millipore). The tanycytes and ependymal cells were labeled with vimentin via immunofluorescent staining (anti-vimentin diluted in 1:400, Abcam). The DR, which is adjacent to the CSF-contacting nucleus, was cut into slices and stained for its specific marker, 5-HT, using immunofluorescence (anti-serotonin diluted in 1:800, Abcam). Group C was the control group and only received CB-HRP microinjection into the ventricle to label the CSF-contacting nucleus. The morphologies of the CSF-contacting nucleus, tanycytes, ependymal cells, ventricle wall surface, and DR in group C was observed at the same manner as for group B.

The immunofluorescent results were captured by confocal laser microscopy (Leica TCS SP2, Germany). The ultrastructural changes of the ventricle wall surface in groups B and C were observed and captured with a scanning electron microscope (Teneo VS, USA).

### Morphological observations after CB-SAP ablation in the CSF-contacting nucleus

The rats were divided into the control group (0 d) and the ablation group. The latter group was further subdivided into ablation groups at 1 d, 3 d, 5 d, 7 d, and 10 d (*n* = 6). Rats were perfused at the indicated time points. Brain segments slightly longer than the CSF-contacting nucleus were acquired on a stereotaxic instrument (Bregma: −7.40 to −9.80 mm). The segments were cut coronally on a cryostat (Leica CM1900, Germany) at 40 μm thickness. All sections underwent CB immunofluorescent staining (anti-CB diluted in 1:600, Merck Millipore). Sections were serially mounted. Morphological changes within the CB-positive neurons were observed by confocal laser microscopy (Leica TCS SP2, Germany). The remnant morphology of the CSF-contacting nucleus at the different times after ablation (0 d, 1 d, 3 d, 5 d, 7 d, and 10 d) was reconstructed spatially with Imaris software, version 8.4.1 (Bitplane, USA). The number of neurons in the CSF-contacting nucleus was counted using Image-Pro Plus 7.0 software.

### Animals' vital status, survival and common physiological evaluation after CB-SAP ablation of the CSF-contacting nucleus

The rats' vital status and survival were recorded for 10 days after CB-SAP microinjection into the lateral ventricle. Baseline scores of the rats' physiological parameters (body weight, respiratory rate, heart rate, and temperature) were measured before the CSF-contacting nucleus ablation. Then, the rats received CB-SAP microinjections into the lateral ventricle. After surgery, the rats were housed with a 12 h light/12 h dark cycle at room temperature (23 ± 1°C) with *ad libitum* access to food and water. The common physiological parameters were measured at days 1, 3, 5, 7, and 10 after the CSF-contacting nucleus ablation (*n* = 6).

### Data analysis

SPSS 13.0 software was used for data analysis. The data are presented as the mean ± standard deviation (SD). The morphological changes of the CSF-contacting nucleus, tanycytes, ependymal cells, and DR in the ablation and control groups were compared with Student's *t*-test. The number of neurons in the CSF-contacting nucleus at different time points after CB-SAP microinjection into the ventricle was analyzed by the Kruskal-Wallis test followed by the Mann-Whitney *U*-test. Any difference with *P* < 0.05 was considered significant.

## Results

### The flow of CB-SAP within the ventricular system

**Figure 1:**
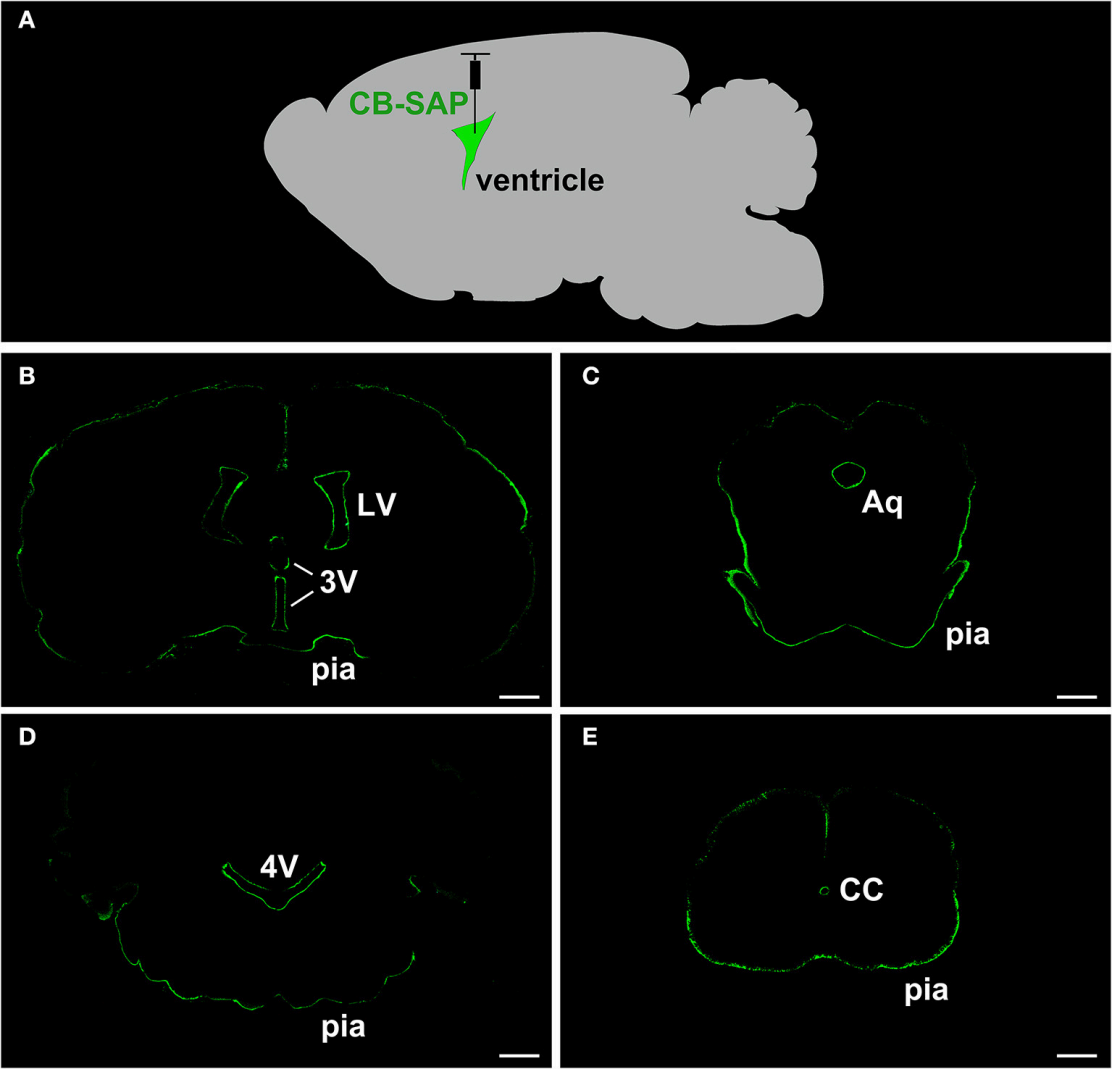
SAP immunofluorescent staining (green) shows a clear outline along the ventricular system. The nearby brain parenchyma displays no immunofluorescent labeling. **(A)** The schematic diagram of CB-SAP microinjection into the lateral ventricle. **(B–E)** SAP immunofluorescent staining along the ventricular system. Bar = 1 mm in **(B–D)**. Bar = 500 μm in **(E)**. LV, lateral ventricle; 3V, 3rd ventricle; Aq, aqueduct; 4V, 4th ventricle; CC, central canal; pia, pia mater.

The CB-SAP distribution was evaluated at nearly 24 h after CB-SAP microinjection into the lateral ventricle. The SAP fluorescent product (green) was confined to the ventricular system. The outlines of the lateral ventricle (LV), the third ventricle (3V), the aqueduct (Aq), the fourth ventricle (4V), the central canal of the spinal cord (CC), and the pia mater of the brain presented clearly. The nearby brain parenchyma had no immunofluorescent labeling (Figure 1).

### The specific distribution of CB-SAP in the CSF-contacting nucleus

**Figure 2:**
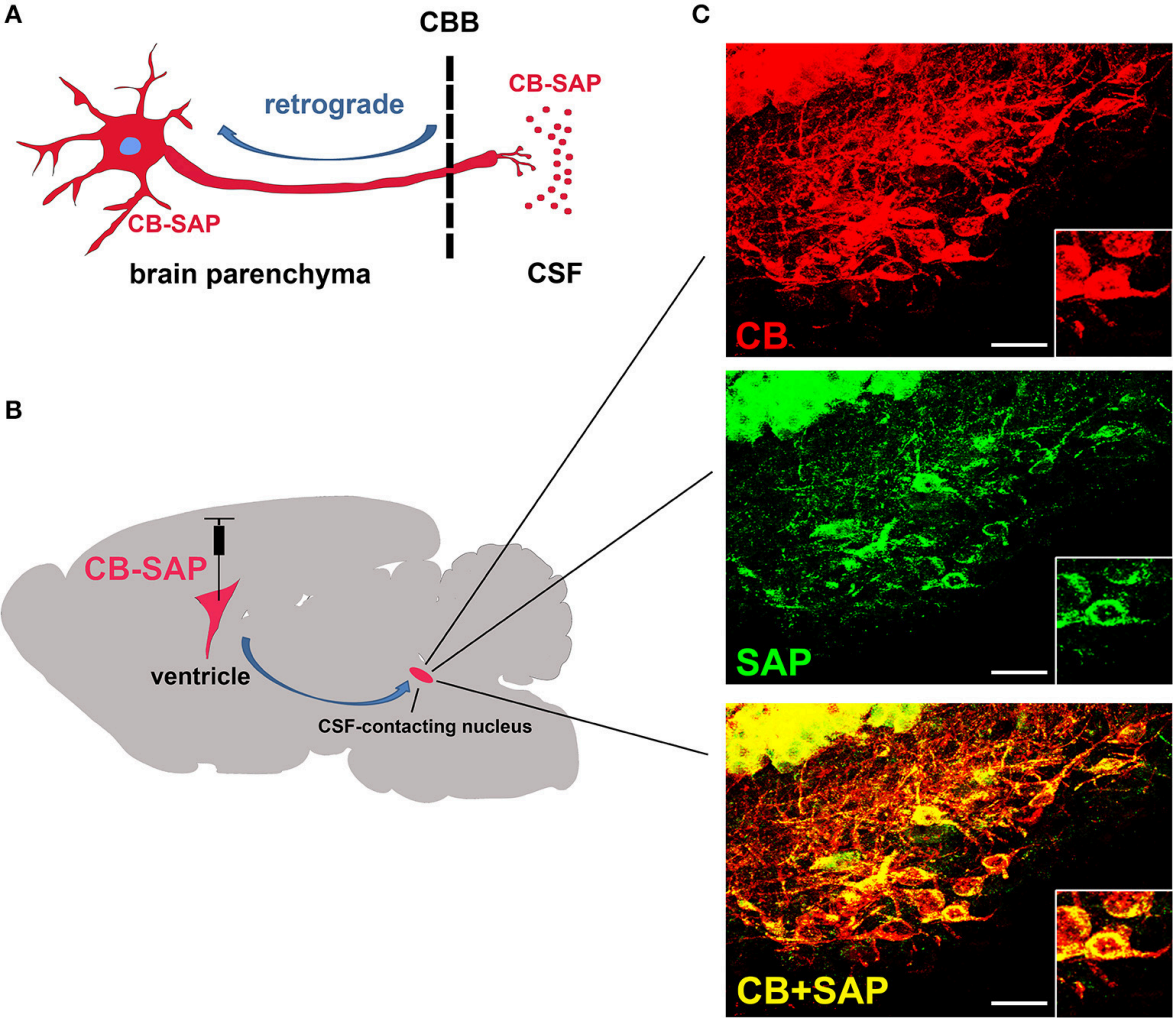
The specific distribution of CB-SAP in the CSF-contacting nucleus. **(A,B)** The schematic diagram of how CB-SAP is transported in retrograde from the CSF to the CSF-contacting nucleus in the parenchyma. **(C)** The CSF-contacting nucleus is labeled for CB (red), SAP (green), and CB/SAP (yellow). The boxed regions show a magnification of three labeled neurons. CBB: cerebrospinal fluid-brain barrier. Bar = 25 μm.

After CB-SAP was microinjected into the ventricle, it was transported in retrograde from the CSF to the CSF-contacting nucleus. Immunofluorescent labeling for neurons containing CB (CSF-contacting nucleus labeling agent), SAP (damaging agent), and CB/SAP (double stained) was found in the same neuron in the CSF-contacting nucleus. Other structures in the brain parenchyma displayed no immunofluorescent labeling (Figure 2).

### CSF-contacting structures within the ventricle wall and the nucleus in the parenchyma adjacent to the CSF-contacting nucleus are not damaged by CB-SAP

The morphology of corresponding structures was assessed at 5 days after CB-SAP microinjection into the lateral ventricle.

**Figure 3:**
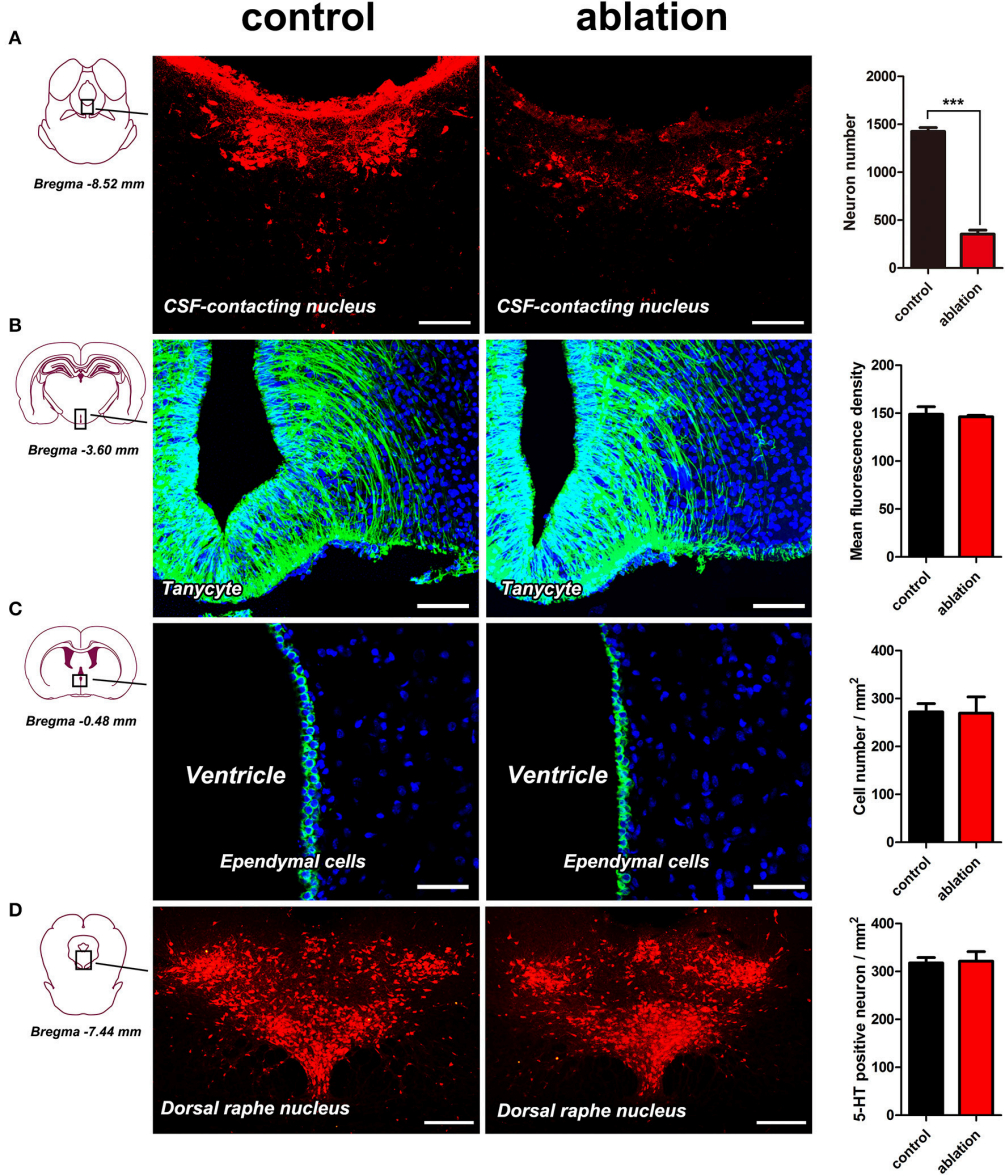
Morphological changes within the CSF-contacting nucleus **(A)**, tanycytes **(B)**, ependymal cells **(C)**, and dorsal raphe nucleus **(D)** after ablation. Bar = 100 μm in **(A,B)**, bar = 50 μm in **(C)**, bar = 250 μm in **(D)**. ^***^P < 0.001.

**Figure 4:**
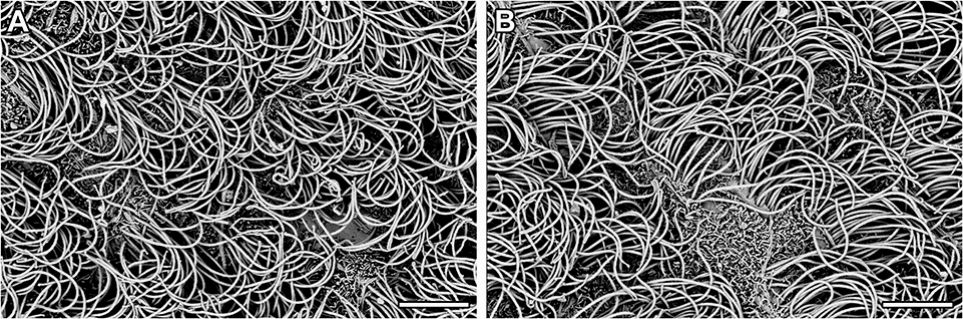
The ventricle wall surface (taken from 3V) in the control group **(A)** and the ablation group **(B)** of rats under a scanning electron microscope. Bar = 5 μm.

1. The neurons in the CSF-contacting nucleus labeled by immunofluorescence for CB were complete and intact in the control group. The neurons in the CSF-contacting nucleus were blurred and damaged significantly [*t*_(4)_ = 31.72, *P* < 0.001] after ablation (Figure 3A).
2. The morphology of tanycytes and ependymal cells within the ventricle walls was not changed significantly [*t*_(4)_ = 0.5658, *P* = 0.60 for tanycytes; *t*_(4)_ = 0.1208, *P* = 0.91 for ependymal cells] after ablation (Figures 3B,C).
3. The dorsal raphe nucleus (DR), the nearest non-CSF-contacting structure adjacent to the CSF-contacting nucleus, was labeled via immunofluorescence for serotonin (5-HT) and was not changed significantly after ablation [*t*_(4)_ = 0.2938, *P* = 0.78] (Figure 3D).
4. The ultrastructures within the ventricle wall that directly contact the CSF were evaluated by scanning electron microscopy after CSF-contacting nucleus ablation. These structures were not changed significantly after ablation when compared with the control group (Figure 4).

### The morphology and number of neurons in the CSF-contacting nucleus after ablation

#### Morphology

**Figure 5:**
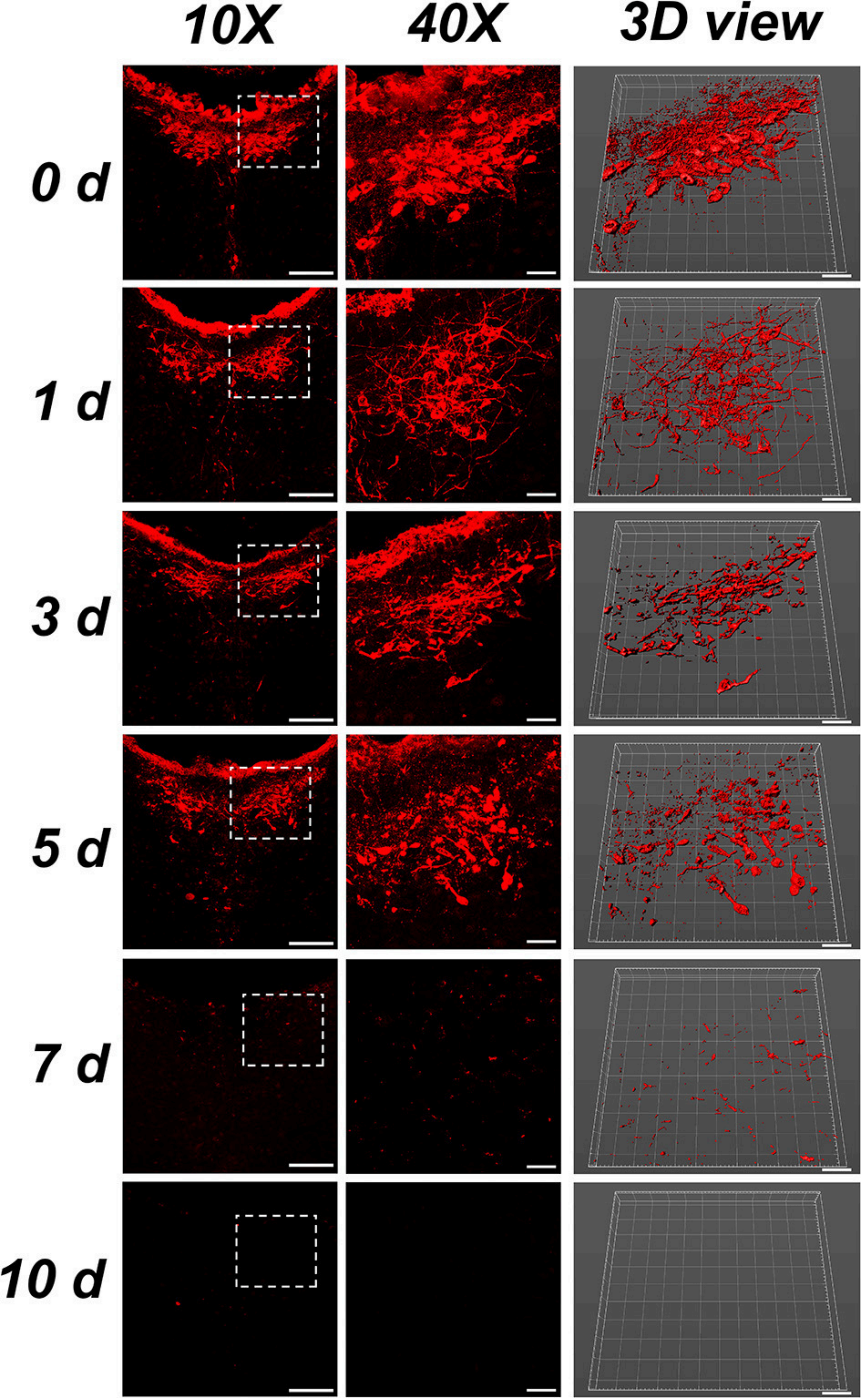
Morphology of the CSF-contacting nucleus at different time points after ablation. Bar = 100 μm in the 10X figures, bar = 30 μm in the 40X and 3D figures. Neurons in 0 d group are the morphology of CB positive neurons in rats' CSF-contacting nucleus before ablation. Neurons in 1,3,5,7 d groups are the morphology of CB positive neurons in rats' CSF-contacting nucleus after ablation.

The neurons in the CSF-contacting nucleus present with CB-positive red fluorescent products. In the control group (0 d), the neurons were intact and regular. The neural processes were clear and dense. On the first day after ablation, the neurons shrank and were irregularly shaped, and the processes became sparse. On the third to fifth days after ablation, neural somas and processes were damaged into fragments. On the seventh day after ablation, only a few fragmented positive structures were detected. On the tenth day after ablation, no positive structures existed in the CSF-contacting nucleus (Figure 5).

#### Total neuron numbers

**Figure 6:**
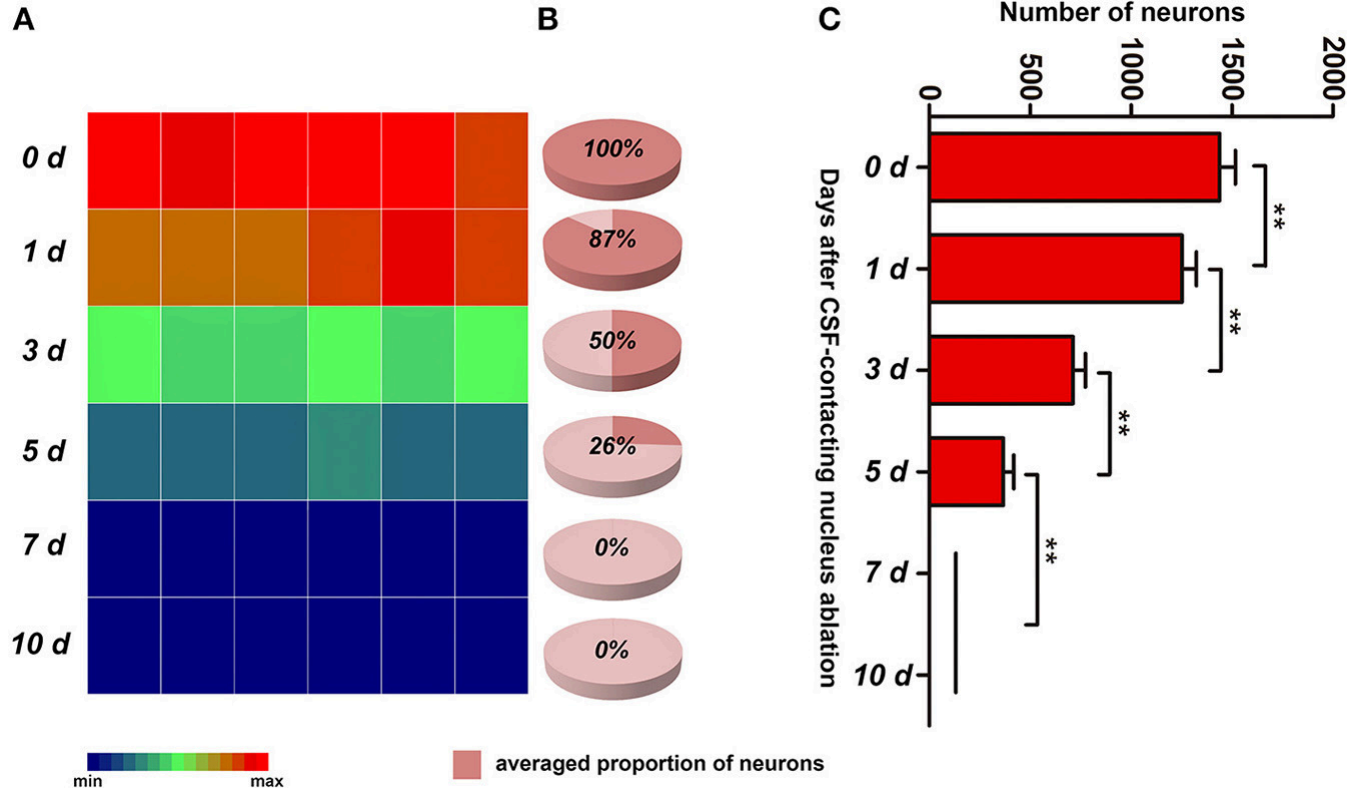
The number of neurons in the CSF-contacting nucleus at different time points after ablation. **(A)** color map, **(B)** pie chart, **(C)** histogram. ^**^P < 0.01. The number of neurons in 0 d group is the CB positive neurons in rats' CSF-contacting nucleus before ablation. The number of neurons in 1, 3, 5, 7 d groups is the CB positive neurons in rats' CSF-contacting nucleus after ablation.

In the control group (0 d), there were 1,439 ± 78 (defined as 100%) neurons in the CSF-contacting nucleus. On the first, third and fifth days after ablation, the numbers of neurons were 1,252 ± 72 (approximately 87%), 651 ± 24 (approximately 50%), and 268 ± 15 (approximately 26%), respectively. On the seventh and tenth days after ablation, no intact neurons existed (0%). The number of neurons in the CSF-contacting nucleus decreased significantly after ablation (χ^2^ = 34.241, *P* < 0.001), and the neuron number reduced significantly compared with that at prior time points (Figure 6).

### Rats' vital status, survival and common physiological parameters after CSF-contacting nucleus ablation

**Figure 7:**
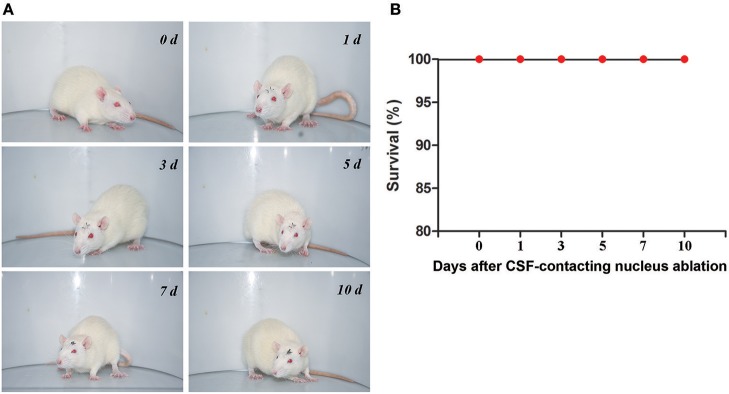
Rats' vital status **(A)** and survival **(B)** at different times after CSF-contacting nucleus ablation.

After CB-SAP microinjections into the lateral ventricles, no obvious effects were observed in the vital status of the animals; likewise, there was no observed influence on the rats' survival during the observation period (Figure 7). The rats' common physiological parameters (body weight, respiratory rate, heart rate, and temperature) remained steady during the experimental period (Table 1).

**Table 1:** Rats' common physiological parameters after CSF-contacting nucleus ablation (*n* = 6, $\bar{x}$ ± s).

**Physiological parameter**	**Day 0**	**Day 1**	**Day 3**	**Day 5**	**Day 7**	**Day 10**
Body weight (g)	306 ± 4	295 ± 8	296 ± 8	298 ± 7	298 ± 6	301 ± 5
Respiratory rate (/min)	94 ± 3	95 ± 2	93 ± 2	95 ± 2	91 ± 4	92 ± 3
Heart rate (/min)	378 ± 25	392 ± 19	385 ± 16	373 ± 20	368 ± 14	375 ± 17
Temperature (°C)	38.2 ± 0.2	38.2 ± 0.6	38.3 ± 0.4	38.4 ± 0.2	38.5 ± 0.3	38.3 ± 0.5

## Discussion

### CB-SAP is an ideal CSF-contacting nucleus ablation agent

Damaging a specific structure in the brain is one of the most essential methods used in neuroscience studies. Using a stereotaxic technique, the chemical degeneration agent (Lanciego et al., 2008; Sheth et al., 2016; Tait et al., 2017) or damaging electrode can be injected or located into the brain to damage the targeted regions (Jung et al., 2016). However, low accuracy and the possibility of damaging nearby structures make these methods controversial. For a damaging agent, avoiding damage to nearby structures not only provides specificity but is also the key in establishing nucleus-deficient model animals.

CB is the non-toxic part of the cholera toxin. It can bind to its receptor and enter into a cell by receptor-mediated internalization. It mainly transports in retrograde from the axon terminals to neural somas and dendrites (Lanciego and Wouterlood, 2011). Previous studies in our research group have confirmed that CB-HRP, which is a tracer used mainly for the peripheral nervous system, can specifically label the CSF-contacting nucleus (Wang and Zhang, 1992; Zhang et al., 1995, 2003; Lu et al., 2008). Zhou et al. further found that a microinjection of CB alone into the ventricular system has a similar labeling effect to CB-HRP (Zhou et al., 2013). Therefore, CB is also regarded as a specific tracer for labeling the CSF-contacting nucleus.

SAP is a type I ribosome-inactivating protein (Thorpe et al., 1985). When SAP couples with a specific ligand or receptor-specific antibody, the latter part of the coupling product can recognize and bind to the epitope of the receptors on the cellar surface. The formed complex induces internalization, thus causing cell death. Using this feature, researchers have recently coupled several chemical groups with SAP for the targeted ablation of specific cell types (Göz et al., 2008; Wiese et al., 2013; Lee et al., 2014).

CB-SAP, the coupled product of the two agents, has mainly been used to damage the peripheral nervous system (Llewellyn-Smith et al., 2000; Nichols et al., 2015). Its application to damaging the CSF-contacting neurons, a recently recognized special nucleus within the brain, has not been reported previously.

In the present study, after CB-SAP was microinjected into the lateral ventricle, it distributed along the surface of the ventricular system and was unable to pass through the brain-CSF barrier. The tracer CB and the degeneration agent SAP co-existed in the same neuron and only damaged the CSF-contacting nucleus. We further evaluated the influence of this ablation method on the structures that directly contacted the CSF, such as tanycytes, ependymal cells, and ultrastructures of the ventricle wall surface and the DR, which is the nearest structure adjacent to the CSF-contacting nucleus in the brain parenchyma. The results demonstrate that only the neurons in the CSF-contacting nucleus are specifically damaged; other structures in the brain are unaffected. Therefore, CB-SAP is an ideal CSF-contacting nucleus ablation agent.

The CSF-contacting nucleus in the central nervous system can be labeled and damaged more preferably by the tracing agent CB-HRP and the degeneration agent CB-SAP. These agents are relatively insensitive to the central nervous system and more commonly used for peripheral nervous system studies which will provide a suppositional space to understand the biological functions of the CSF-contacting nucleus.

### The establishment of the CSF-contacting nucleus “knockout” model animal

Because CB-SAP is an ideal CSF-contacting nucleus ablation agent, we further investigated the ablation regularity of CB-SAP toward the CSF-contacting nucleus. The observation that CB-SAP damaged the entire CSF-contacting nucleus after a consistent length of time was the key parameter for establishing the CSF-contacting nucleus “knockout” model animal.

The number of neurons in the CSF-contacting nucleus was evaluated at days 1–10 after CB-SAP microinjections into the lateral ventricles to determine the temporal pattern of the ablation. Over time, the lesion to the CSF-contacting nucleus aggravated gradually. The CSF-contacting nucleus was entirely damaged by the seventh day after ablation. The results not only provided a reference for the CSF-contacting nucleus ablation at different times but also clarified that a complete CSF-contacting nucleus “knockout” model animal was generated at seven days after CB-SAP microinjection into the ventricular system.

As a model animal, a good vital status, low death rates, and steady common physiological parameters are important for further mechanism studies. For example, some molecules are essential for the animals' development and living. After knockout of the corresponding genes, animals cannot survive to adulthood. In the present study, the CSF-contacting nucleus ablation did not cause the animals' death. The animals survived throughout the study period, and the ablation had few effects on the animals' common physiological parameters (including body weight, respiratory rate, heart rate, and temperature). Therefore, the model animal was considered stable and reliable.

### Applications

Model animals are indispensable tools in scientific studies. They provide a method for studying certain biological functions. The CSF-contacting nucleus is a special nucleus within the brain that has been recognized in recent years. Its anatomical structure is unique among the already known functional structures in the brain. Its neural somas are located in the parenchyma and communicate with other structures, such as non-CSF-contacting neurons, glia cells, and blood vessels in the brain. The processes have sensing, secreting and releasing functions and stretch directly into the CSF. Uncovering its functions will certainly help interpret the information transmission between the brain and body fluid with respect to the existence of the barriers in the brain (Liddelow, 2011) and their roles in complicated physiological and pathological situations.

Many mysteries remain regarding this special nucleus. In our previous studies, we have studied the distribution and change of dozens of neuroactive substances (including neurotransmitters, receptors and ion channel proteins) in the CSF-contacting nucleus under inflammatory pain (Wang et al., 2014), neuropathic pain (Li et al., 2015; Wang et al., 2015), and visceral pain (Zhang et al., 2017), etc. The exact roles of the CSF-contacting nucleus in pain and other physiological as well as pathological conditions are still unclear. Therefore, it is essential to establish a dedicated model animal for studying this nucleus. Because this particular nucleus has only been recognized in recent years, no model animals are available specifically for this nucleus. The present study provides an model animal similar to a gene knockout model animal. The functions of the CSF-contacting nucleus can be tested extensively under different situations using this model animal. The method is specific, authentic, scientific and easy to operate. It avoids the drawbacks of the ablation of a specific nucleus, such as potential low targeting accuracy and the possibility of damaging nearby structures. The CSF-contacting nucleus “knockout” model animal was successfully established and can be used by interested researchers.

In summary, 7 days after the CB-SAP microinjection into the ventricular system, all neurons in the CSF-contacting nucleus are specifically damaged, thus successfully establishing a CSF-contacting nucleus “knockout” model animal.

## Methodological overview

Sprague-Dawley rats of both sexes, with weights of approximately 300 g, received a 3 μl CB-SAP (ATS, USA) [500 ng, dissolved in PBS (0.01 M, pH 7.4)] microinjection into the lateral ventricle according to the stereotaxic coordinates provided by Paxinos and Watson (2007). The injections into the lateral ventricle were confirmed by successful extraction of the CSF. After animals survived at least 7 days, the CSF-contacting nucleus was entirely damaged, and the CSF-contacting nucleus “knockout” model animals were successfully established.

## Author contributions

S-YS conducted the studies. L-CZ and S-YS designed the study and prepared the manuscript. All authors read and approved the manuscript.

### Conflict of interest statement

The authors declare that the research was conducted in the absence of any commercial or financial relationships that could be construed as a potential conflict of interest.
